# Predegenerated Schwann cells–a novel prospect for cell therapy for glaucoma: neuroprotection, neuroregeneration and neuroplasticity

**DOI:** 10.1038/srep23187

**Published:** 2016-04-01

**Authors:** Adrian Smedowski, Xiaonan Liu, Marita Pietrucha-Dutczak, Iwona Matuszek, Markku Varjosalo, Joanna Lewin-Kowalik

**Affiliations:** 1 Chair and Department of Physiology, School of Medicine in Katowice, Medical University of Silesia, Medykow 18, 40-752 Katowice, Poland; 2 Department of Ophthalmology, University of Eastern Finland, P.O. Box 1627, 70211 Kuopio, Finland; 3 Department of Ophthalmology, School of Medicine with the Division of Dentistry in Zabrze, Medical University of Silesia, Panewnicka 65, 40-760 Katowice, Poland; 4 Institute of Biotechnology, P.O. Box 65, University of Helsinki, 00014 Helsinki, Finland

## Abstract

Glaucoma is an optic neuropathy that leads to irreversible blindness. Because the current therapies are not sufficient to protect against glaucoma-induced visual impairment, new treatment approaches are necessary to prevent disease progression. Cell transplantation techniques are currently considered to be among the most promising opportunities for nervous system damage treatment. The beneficial effects of undifferentiated cells have been investigated in experimental models of glaucoma, however experiments were accompanied by various barriers, which would make putative treatment difficult or even impossible to apply in a clinical setting. The novel therapy proposed in our study creates conditions to eliminate some of the identified barriers described for precursor cells transplantation and allows us to observe direct neuroprotective and pro-regenerative effects in ongoing optic neuropathy without additional modifications to the transplanted cells. We demonstrated that the proposed novel Schwann cell therapy might be promising, effective and easy to apply, and is safer than the alternative cell therapies for the treatment of glaucoma.

Glaucoma is an optic neuropathy that leads to the continuous and progressive destruction of retinal ganglion cells (RGC), whose axons form the optic nerve, and finally, to blindness^1^,^2^,^3^. The association between glaucoma development and increased intraocular pressure (IOP), the basic measurable pathogenic factor, varies worldwide and occurs clinically with higher frequency in Western countries than in Asian populations, however this is not the only identified risk factor of the neuropathy^4^,^5^,^6^,^7^,^8^. Since current therapeutic strategies, i.e. pharmacological and surgical approaches targeting increased IOP, are not sufficient enough to protect against glaucoma blindness, and to restore the function of already injured RGC, new effective therapeutic strategies focused on RGC neuroprotection and their regeneration are expected to be developed^9^.

Cell transplantation techniques, applying various types of stem and progenitor cells, are currently considered to be a very promising tool in advanced therapies for central nervous system (CNS) damage, including damage to the retina and optic nerve; however, many obstacles for their usage in the retina have already been described^10^,^11^,^12^,^13^,^14^,^15^,^16^. Concerning cell transplantation to the inner retina, there are two directions these therapies might take: RGC neuroprotection and RGC replacement^17^. In most studies of glaucoma cell therapies, only stem and progenitor cells are considered, and no prospects for mature, differentiated cell usage are discussed in recent reviews^16^,^17^,^18^.

Schwann cells (SC) are the major glial cells in the peripheral nervous system. They are capable of stimulating the regeneration of both the peripheral and central nervous systems^19^. SC-induced regeneration manifests in the generation of new axons as well as the branching of already existing ones^20^. There are several possibilities to activate SC under various conditions such as *in vivo* predegeneration, which can last various amounts of time, or glucose-dependent *in vitro* activation; however, *in situ* 7-day nerve predegeneration, which occurs *in vivo* as a result of peripheral nerve injury, has been claimed to be the most effective^21^,^22^,^23^,^24^. After nerve injury, SC create an environment favorable to the spontaneous regeneration of axons due to secretion of adhesion molecules and various trophic factors; SC obtained from the injured nerve in this time-window (i.e., after 7 days) are highly active and viable^25^,^26^,^27^.

In the present study, based on experience and promising results of SC transplantations in different CNS injuries, we introduced, for the first time, the allotransplantation of adult, differentiated SCs in a chronic, glaucomatous optic nerve neuropathy. In the reference group, we generated an acute optic nerve neuropathy (i.e., optic nerve crush, ONC); additionally, we cultured *ex vivo* retinal explants. Our aim was to detect potential neuroprotective and pro-regenerative effects of applied SC therapy toward RGC under experimental conditions in chronic and acute optic neuropathy. We also considered the safety of the applied therapy and its potential future utility in clinical applications.

## Results

### SC’s secretome and SC’s homogenate does not contain neurotrophic factors

**Figure 1:**
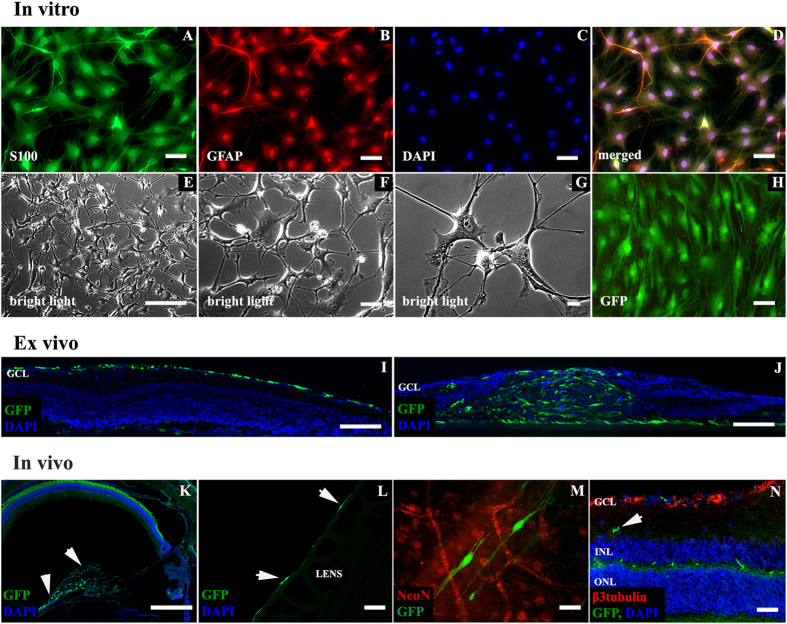
Schwann cells in *in vitro, ex vivo* and *in vivo* conditions. (**A–H**) – immunofluorescent characterization (**A–D**), SC in culture (**E–G**) and GFP expression after Lv-eGFP transduction (**H**). Scale bar = 50 μm (**A–D,H**); 500 μm (**E**); 100 μm (**F**); 20 μm (**G**). (**I–J**) – *ex vivo* retinal explants. (**I**) – SC cultured with explants with intact ILM are covering retinal surfaces with no signs of intraretinal penetration. (**J**) – mechanical interruption of the ILM resulted in massive infiltration of retinal tissue by SC accompanied by damage of lamellar retinal architecture. Scale bar = 100 μm (**I,J**). (**K–N**) – localization of SC after intravitreal transplantation *in vivo*. SC (arrows) detected as a bolus in vitreal space (**K**) on posterior lens surface (**L**) some of SC were integrated with retinal surface (**M**). Only single SC were observed in deeper retinal layers (**N**, arrow). Scale bar = 500 μm (**K**); 50 μm (**L–N**). GCL- ganglion cell layer; INL – inner nuclear layer; ONL – outer nuclear layer.

To evaluate purity of SC culture, we calculated the ratio of cells that were co-localized for the S100 protein and glial fibrillary acidic protein (GFAP) in relation to those that were DAPI counterstained for cell nuclei, this ratio was about 99–100% (Fig. 1A–H). To confirm proteomic features of cultivated SC, culture medium samples and SC homogenate were analyzed by mass spectrometry (MS). The most strongly represented components of SC proteome consisted of extracellular matrix components, adhesion molecules, growth factor binding proteins, ion channel modulators and proteins involved in antioxidant cell protection, neuronal cells growth and axonal development (see Supplementary Table 1). Other growth-related factors such as nerve growth factor (NGF), brain derived neurotrophic factor (BDNF), ciliary neurotrophic factor (CNTF) and neurotrophin 3 (NT3), which are widely described as characteristic of SC, were not detected. Positive controls demonstrated the ability to detect low concentrations of BDNF and CNTF in culture medium using MS.

### Intraretinal migration of transplanted SC is associated with disruption of retinal architecture

We tested migration capacity of transplanted SC in *ex vivo* and *in vivo* conditions. In *ex vivo* retinal insert explants, localization of transplanted SCs depended on the inner limiting membrane (ILM) continuity. In explants with intact ILM, SC were located on explant surface, and only single cells were able to penetrate inside the retinal tissue (Fig. 1I). In explants with an interrupted ILM, massive infiltration of retinal tissue by SC was observed. This migration was associated with destruction of the lamellar structure of retina (Fig. 1J). *In vivo* transplanted SC were located mostly in the vitreous body, on the posterior lens capsule and on the retinal surface (Fig. 1K–M). In *in vivo* samples, only single cells were detected within deeper retinal layers (Fig. 1N, arrow). Transplanted SC demonstrated preserved typical cell morphology in *in vivo* conditions (Fig. 1M).

### Transplanted SC prolong RGC survival in *ex vivo* retinal explants

**Figure 2:**
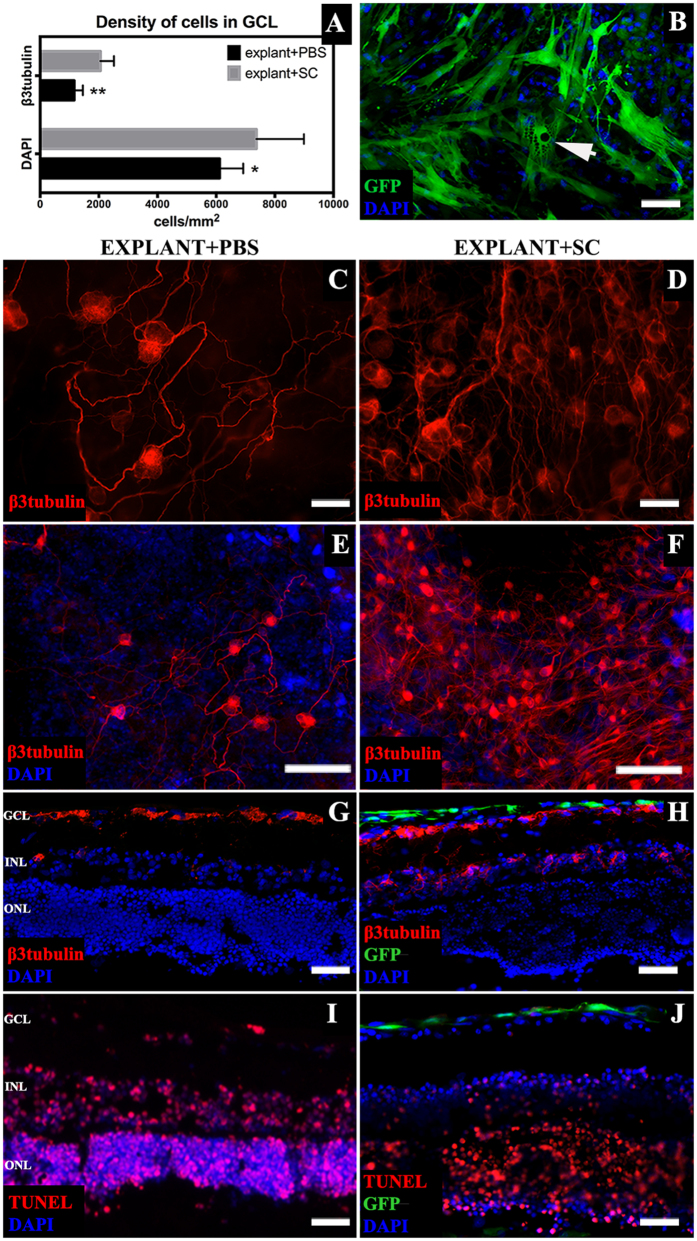
*Ex vivo* insert retinal explants characterization. (**A**) – density of cells in the GCL of explants cultured with SC suspension and PBS; SC treatment resulted with higher density of total cells in GCL as well as β3tubulin positive RGC. (**B)** – SC on retinal explant surface with visible granular intracellular structure (arrow). (**C–H**) – β3tubulin-positive cells in the GCL of explants treated with SC or PBS (**C–F**) – whole mounted explants; (**G,H**) – retinal cross-sections). (**I,J**) – TUNEL staining of explants cross cryosections revealed a decreased number of apoptotic cells in the SC surroundings (**J**). Scale bar = 50 μm (**B–D,G–J**); scale bar = 500 μm (**E,F**). GCL- ganglion cell layer; INL – inner nuclear layer; ONL – outer nuclear layer, SC – Schwann cells.

In retinal insert explants co-cultured with SC and with intact ILM, the density of β3tubulin-positive cells in the ganglion cell layer (GCL) after 10 days was significantly higher (2054 ± 461 cells) than in PBS-treated explants (1163 ± 295 cells) (Mann-Whitney U-test, p < 0.01) (Fig. 2A,C–H). SC cytoplasm on explant surfaces developed granular structure, a possible sign of secretory activity (Fig. 2B, arrow). In explants with interrupted ILM, identification of GCL was impossible due to the loss of the lamellar retinal structure (Fig. 1J). The presence of SC on the explant surface visibly reduced the number of apoptotic cells within inner retinal layers (Fig. 2I,J).

### Evaluation of IOP in experimental glaucoma model

**Figure 3:**
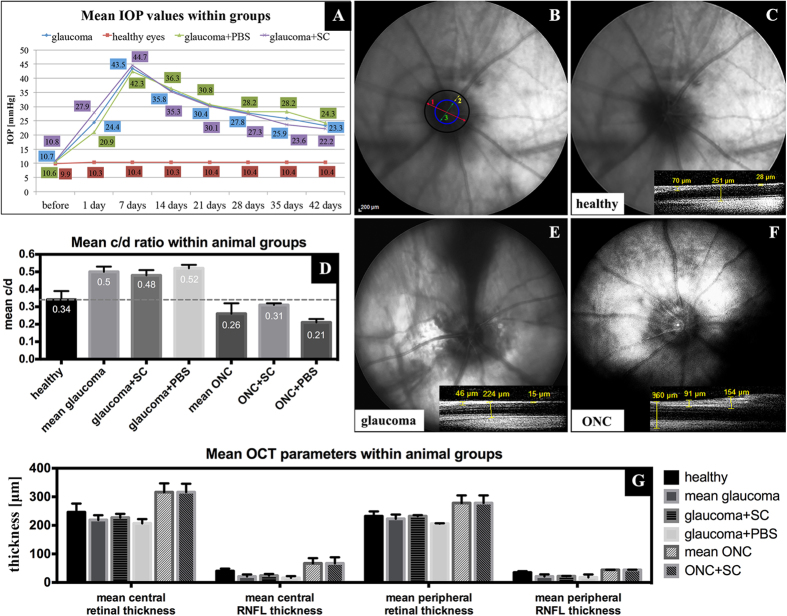
Mean IOP values in experimental glaucoma groups (**A**); stable elevated IOP with no differences between treated and control groups ensures “pressure independent” protective effects towards RGC. Examples of eye fundus evaluation (B-G). B – a schematic anatomical characterization of optic nerve disc in fundus photograph; 1-optic nerve disc diameter (red); 2-neuroretinal ring diameter (yellow); 3-optic nerve cup diameter (green); cup/disc (c/d) ratio=3/1; for healthy eyes c/d ratio~0.3. C-F – example fundus photographs and c/d ratio in healthy, glaucomatous and ONC eyes; small OCT scans represent examples of retinal thickness in parapapillary area. Increased c/d ratio in glaucomatous eyes due to a thinning of the neuroretinal rim and a decreased c/d ratio in ONC eyes due to retinal nerve fiber edema. G - OCT retinal parameters in healthy, glaucomatous and ONC eyes. In the ONC group with intravitreal PBS injection, insight into eye fundus was very limited and capture of OCT scans was impossible. A statistical comparison between groups of animals was not perform due to the small sample size. ONC - optic nerve crush; SC - Schwann cells.

The mean 6-week IOP in ocular hypertensive eyes was significantly higher than in healthy contralateral eyes (mean ± SD, 31.02 ± 5.5 mmHg and 10.32 ± 0.54 mmHg, respectively, Wilcoxon paired test, p < 0.05). There was no difference between the mean IOP or the IOPs from each time-point when comparing ocular hypertensive eyes treated with SC and those treated with intravitreal PBS injection (Mann-Whitney U-test, p > 0.1) (Fig. 3A).

### Optic nerve disc cupping in eyes with acute and chronic optic neuropathy

In fundus pictures, as shown before, we reported an increased cup/disc (c/d) ratio in glaucomatous eyes due to a thinning of the neuroretinal rim and a decreased c/d ratio in ONC eyes due to retinal nerve fiber edema (Fig. 3B–F). In the ONC control group (with intravitreal PBS injection), insight into eye fundus was very limited due to presence of exudation and capture of OCT scans was impossible. In OCT scans, retinal thinning in the glaucoma group (i.e., due to atrophy) and thickening in the optic nerve crush group (i.e., due to edema) were observed (Fig. 3G). A statistical comparison between SC treated and untreated animals was unable to perform due to the small sample size; however, for the glaucomatous eyes treated with SC optic nerve disc cupping, which is considered to be the most frequently used, tended to be slightly lower than in PBS injected eyes (Fig. 3D).

### RGC bodies and optic nerve axons survival in experimental glaucoma and ONC is improved after SC transplantation

**Figure 4:**
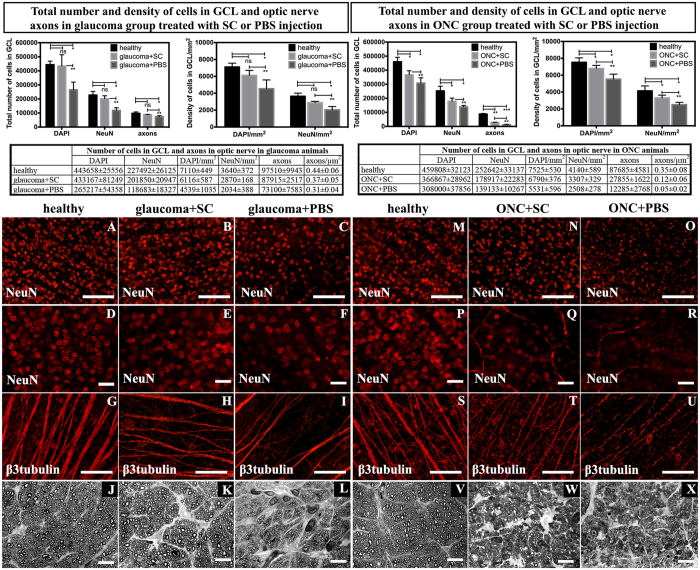
Total number and density of DAPI- and NeuN-positive cells in the GCL and optic nerve axons in glaucoma and ONC eyes. The upper panel contains graphs presenting exact quantitative analysis and statistical comparisons. SC therapy showed neuroprotective activity towards RGC when compared with control PBS injection. A single asterisk indicates statistical significance at the level of p < 0.05, double asterisk p < 0.01 and triple asterisk p < 0.001. (**A–I**, **M–U**) – immunostainings of whole mounted retinas showing GCL cells positive for NeuN with visible reduced cells density within PBS-treated retinas (**A–F** in glaucoma group; **M**–**R** in ONC group) and β3tubulin with marked lower density and thinning of RGC axons (**G–I** in glaucoma group and **S**–**U** in ONC group). (**J–L), (V–X**) – optic nerve cross-sections in glaucoma and ONC; optic nerves from PBS-treated groups showed severe changes including gliosis, axons swelling and macrophages infiltration, while SC-treated groups presented only mild changes in glaucomatous optic nerves and moderate injury after ONC. Scale bar = 50 μm (**D–F), (P–R**); 500 μm (**A–C), (M–O), (G–I), (S–U**); 10 μm (**J–L), (V–X**) SC – Schwann cells; ONC – optic nerve crush.

We hypothesized that intravitreal transplantation of predegenerated SC prevents RGC death in optic neuropathies. In the experimental glaucoma group, there were significant differences in the total number of cell bodies in GCL and their density between SC-treated and PBS-treated eyes (Mann-Whitney U-test, p < 0.01), as well as healthy control and PBS treated eyes (Wilcoxon paired test, p < 0.03); surprisingly, there was no significant difference between healthy control and SC-treated eyes (Wilcoxon paired test, p > 0.1) (Fig. 4 top row graphs, A–I). In the ONC group, significant differences in the total number of GCL cell bodies and density were observed between all groups: SC-treated vs PBS-treated eyes (Mann-Whitney U-test, p < 0.01) and healthy control eyes vs SC-treated and vs PBS-treated eyes (Wilcoxon paired test, p < 0.03 and p < 0.03, respectively) (Fig. 4 top row graphs, M–U). Regarding optic nerve axons, there were differences in the total number and density of axons, similar to the differences noted in GCL cell bodies (SC-treated vs PBS-treated, healthy control eyes vs PBS-treated, and healthy control vs SC-treated (Mann-Whitney U-test, p < 0.01; p < 0.03 and p > 0.1, respectively, in the glaucoma group; and Mann-Whitney U-test, p < 0.01; p < 0.01 and p < 0.01, respectively, in the ONC group) (Fig. 4 top row graphs, J–L, V–X).

Based on retinal histology, to detect inflammatory responses in retina after SC transplantation (concerning safety of SC usage), we performed immunostainings against Iba-1 for microglial cells (including macrophages) and GFAP for glial cells. In these stainings, we did not observe any signs of exacerbation of glial or microglial cell infiltration in groups treated with SC (Supplementary Fig. 1A–J). In optic nerve longitudinal sections, SC treatment prevented the loss of the linear nerve architecture that was associated with a visibly delayed thinning of nerve fibers and reduced proliferation of glial cells (Supplementary Fig. 1K–T).

### SC therapy induces neuroregeneration and RGC neurite outgrowth in *ex vivo* and *in vivo* conditions

**Figure 5:**
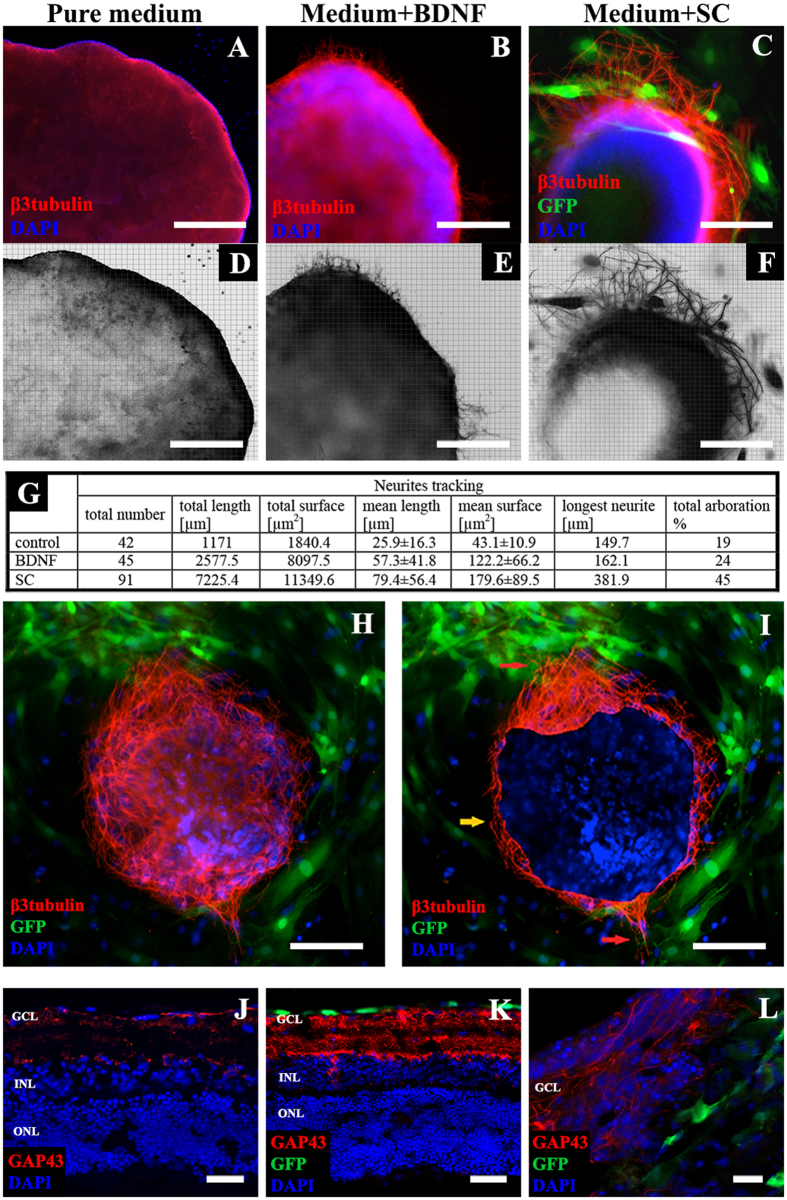
Neurite outgrowths in *ex vivo* retinal explants. (**A–F**) – color and reconstruction pictures of β3tubulin-positive neurites, the most intense outgrowth was observed in SC-treated group, scale bar = 500 μm. (**G)** – quantitative stereological analysis of neurite outgrowths, SC significantly increased number, length and surface of growing neurites. (**H)** – positive tropism of neurite outgrowths in retinal explants culture in the direction of SC. (**I**) – real area of retinal explant labeled with DAPI (to improve interpretation), red arrows indicate intense neurite outgrowth in explant’s areas surrounded by SC, while areas with SC absence (marked with yellow arrow) showed visibly poorer outgrowth, scale bar = 500 μm. (**J–L**) – GAP43 immunostaining in insert retinal explants (cross-cryosections); in explants treated with PBS (**J**) decreased expression of GAP43 is visible. Under SC treatment, there is visible overexpression of a regeneration marker (**K**). L – growing axons within GCL. Scale bar = 50 μm (**J,K**); 20 μm (**L**). GCL- ganglion cell layer; INL – inner nuclear layer; ONL – outer nuclear layer. SC – Schwann cells; BDNF – Brain Derived Neurotrophic Factor.

**Figure 6:**
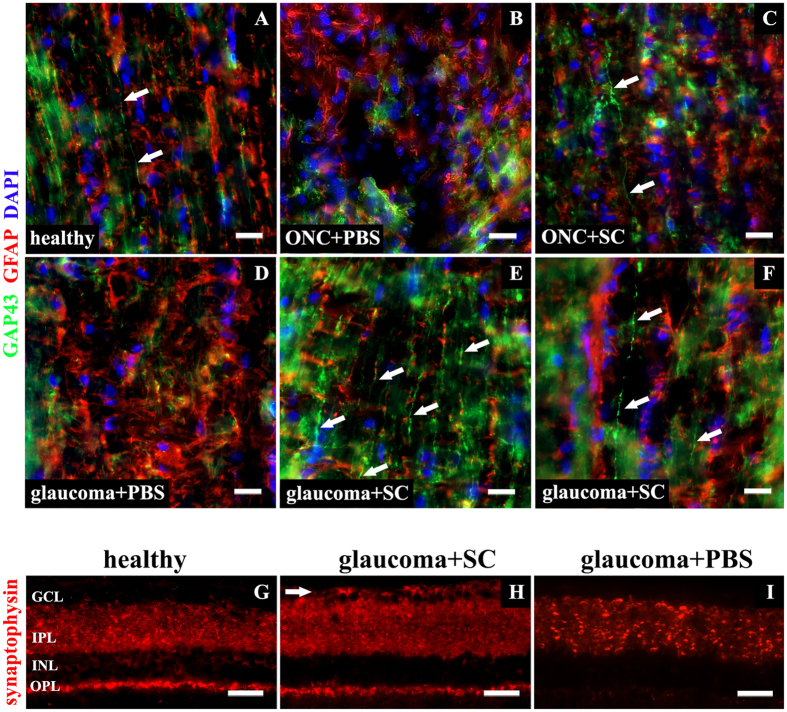
Neuroregeneration and neuroplasticity *in vivo*. (**A–F**) – longitudinal sections of retrobulbar, unmyelinated optic nerve portion. (**A)** – single GAP43-positive fibers in healthy optic nerve (arrows); (**B**) – ONC treated with PBS injection, no visible neurites outgrowth; (**C**) – ONC treated with SC injection with single GAP43-positive fiber visible (arrow); (**D**) – glaucoma treated with PBS injection, no visible neurites outgrowth; (**E–F**) – glaucoma treated with SC injection, numerous, parallel GAP43-positive fibers detected within optic nerve (arrows). Samples were additionally stained for glial marker – GFAP to confirm presence of optic nerve reactive gliosis accompanying damage. Scale bar = 20 μm. (**G–I**) – retinal cross-sections presenting different pattern of synaptophysin expression within retina. In healthy retinas (**G**) synaptophysin expression was visible mostly within IPL and OPL, additionally perinuclear staining was visible in INL and punctate in GCL. Glaucomatous retinas treated with SC-injection (**H**) presented increased expression of synaptophysin, particularly in IPL. Additionally, strong expression of this synaptic marker was visible in the GCL (arrow). In glaucoma samples treated with PBS, decreased expression of synaptophysin within the IPL and outer plexiform layer OPL was observed (**I**). Scale bar = 50 μm. GCL - ganglion cell layer; INL - inner nuclear layer; IPL - inner plexiform layer; OPL - outer plexiform layer. SC – Schwann cells; ONC – optic nerve crush.

Since we showed that transplantation of SC might act neuroprotective towards RGC, the next analyzes focused on neurite outgrowth detection. In reversed P2 retinal explants, there were significant differences among all groups (i.e., control, BDNF- and SC-treated) with respect to the mean and total length and surface area of neurites, as well as the number and arboration of neurites (Mann-Whitney U-test, p < 0.01) (Fig. 5A–G). Neurite outgrowths exhibited a positive tropism that depended on SC localization (Fig. 5H). On Fig. 5I, real area of retinal explant, labeled with DAPI, is marked. Red arrows indicate intense neurite outgrowth in explant’s areas surrounded by SC, while areas with SC absent (marked with yellow arrow) showed visibly poorer outgrowth (Fig. 5I). SC-induced neurite outgrowths (i.e., positive for anti-GAP-43) were also observed in insert retinal explants (Fig. 5J–L). In *in vivo* experiments of both glaucoma and ONC treated with SC, GAP-43 positive parallel nerve fiber growth was detected within retrobulbar, unmyelinated parts of optic nerves; this outgrowth was absent in PBS-treated eyes (Fig. 6A–F).

### SC-induced synaptic plasticity in experimental glaucoma

**Figure 7:**
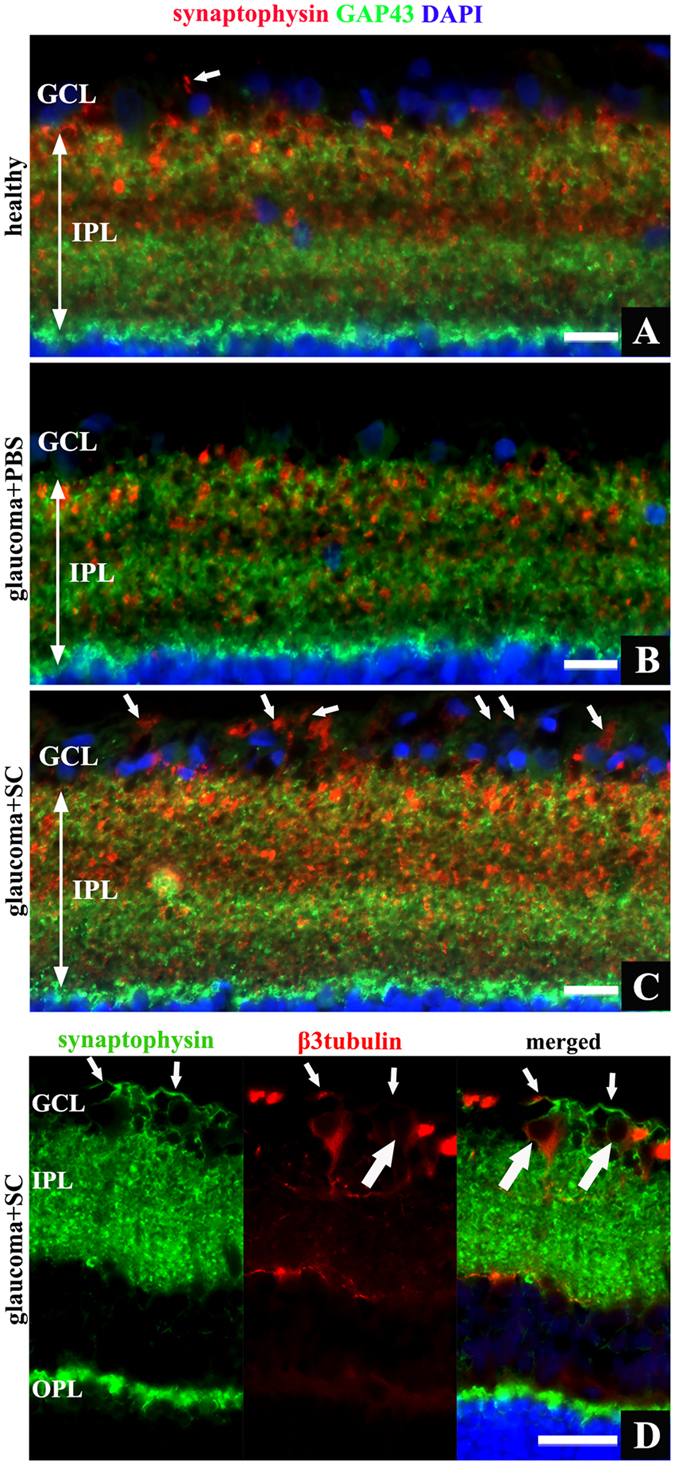
Retinal cross-sections, different expression pattern of GAP43 and synaptophysin proteins within inner retina. (**A)** – healthy retina, high synaptophysin expression visible in IPL and single punctate staining in GCL (arrow). (**B**) – glaucomatous retina treated with PBS revealed decreased synaptophysin expression within IPL. (**C**) – glaucomatous retina treated with SC, strong synaptophysin staining visible in IPL, additionally intense lineal signal detected in GCL – representation of axonal pattern of synaptophysin (arrows). (**D**) – merged image of staining for synaptophysin and β3tubulin revealed that lineal expression of synaptophysin extends in RGC axons (small arrows) projecting from RGC bodies (big arrows). Scale bar = 20 μm (**A–C**), 50 μm (**D**). OPL - outer plexiform layer; IPL - inner plexiform layer; GCL - ganglion cell layer; SC - Schwann cells.

To detect if SC-induced neurites outgrowth is associated with increase of synaptic proteins expression, *in vivo* glaucoma samples were evaluated for expression of synaptic marker — synaptophysin — using immunofluorescence staining (Fig. 6G–I). In glaucomatous retinas treated with PBS-injection, decreased expression of synaptophysin within the inner plexiform layer (IPL) and outer plexiform layer (OPL) was observed (Fig. 6I). SC treatment increased expression of synaptophysin, particularly in IPL (i.e., in afferent synapses between RGC and bipolar cells). Additionally, synaptic marker expression in the GCL (that was absent even in healthy retinas) was visible (Fig. 6H). In SC-treated glaucoma samples, presence of increased retinal expression of synaptophysin, especially this visible in initial part of ganglion cells axons (RNFL) (Fig. 7C,D, small arrows; big arrows on panel D indicate RGC bodies), might be linked with accelerated axonal transport of this synaptic protein to newly grown projections that in theory should tend to create synaptic connections with their proper target. In healthy conditions, where neurites outgrowth within optic nerve was only accidentally seen, synaptophysin expression in GCL was visible only as single punctate staining (Fig. 7A, arrow). In PBS-treated glaucoma samples, where we did not see any signs of neurites outgrowth, also synaptophysin expression within GCL was absent (Fig. 7B).

## Discussion

### Directions in the development of cell therapies for glaucoma

Cell transplantation techniques are currently considered to be one of the most promising treatment options for CNS damage. The most studied direction of cell-based therapies in experimental ophthalmology is the transplantation of cells that, after adaptation to the environment of the eyeball, are designed to release factors affecting retinal neurons’ survival^18^. The other branch of ocular cell therapies, neuronal replacement, remains almost unachievable, mostly due to poor integration of grafted cells^28^,^29^,^30^,^31^,^32^. In recent years, idea of cell transplantation has been successfully introduced into experimental models of ocular hypertension, to study neuroprotection of RGC^10^,^11^,^12^,^15^. However, these studies are employing glaucoma models based on ocular hypertension, it would be also important to investigate neuroprotective effects of cell therapies in models mimicking normal-tension glaucoma^33^.

### Placement of SC therapies for optic neuropathies

Based on a comparison of various cell populations, predominantly precursor cells, regarding the possibility of adapting them for use in spinal cord, hippocampus, retina and optic nerve diseases, SC appear to be particularly useful^34^,^35^. The idea of predegenerated SC therapy for glaucoma was based on available knowledge on CNS and PNS regeneration studies using peripheral nerve predegeneration products, as well as SC in various animal models^36^,^37^,^38^,^39^,^40^,^41^,^42^,^43^. Thus far, SC activity was tested only in models of acute neuropathy — mostly optic nerve transection, in which transplanted SC or nerve predegeneration products improved the survival of RGC as well as promoted neurite outgrowths^39^,^44^,^45^.

### Suspected mechanism of SC activity do not include neurotrophic factors

Although the observed effect of SC grafting and the safety and appropriateness of its use seems to not raise doubts, it is challenging to explain the exact mechanisms of SC activity in our study. The positive neuroprotective and pro-regenerative effect of activated SC therapy theoretically steps from three main groups of factors secreted by these cells, similar as described for mesenchymal stem cells, oligodendrocyte progenitor cells, Müller precursors and olfactory glial cells^10^,^11^,^12^,^15^. Among the factors that are identified as potentially important in regeneration, neurotrophic factors and their receptors (i.e., NGF, BDNF, CNTF, NT3), extracellular matrix components and both adhesion and membrane proteins have been described^25^,^26^,^27^,^46^. An additional, potentially important mechanism is increased sensitivity of receptors to growth factors, which appears to be a result of barotrauma in the retina and optic nerve head^47^. In theory factors secreted by SC likely have synergistic effects as evidenced by the observation that administration of neurotrophic factors by themselves did not have as many beneficial effects as cell therapy^47^,^48^. In the current literature, contact between the membrane of SC and axons is described as an important factor that may be involved in axonal regrowth, however in our case, with limited intraretinal cell migration, this condition seems not to be so significant^49^. To describe characteristics of cultivated SC and identify potential mechanisms of interactions with the retina, we performed proteomic analysis of the culture medium and SC homogenate. It is known that SC are a rich source of many biological agents that might be released into the extracellular space, affecting neighboring cells, other membrane-associated proteins are primarily responsible for the autocrine or paracrine regulation of SC themselves^50^,^51^. Proteomics of cell-free culture medium and of SC homogenate confirmed most of their known proteomic features and revealed the presence of extracellular matrix proteins, adhesion molecules, proteins associated with growth factors (i.e., their receptors and binding proteins), membrane proteins and mitochondrial enzymes; however, the expected presence of the neurotrophic factors (i.e., NGF, BDNF, CNTF, NT3) has not been confirmed, which was highly suprising. Additionally, MS analysis revealed the presence of a large group of proteins involved in cells’ oxidative stress defense and LRP2 receptor agonists (involved in neuronal development and axonal growth), which we suspect to be key mechanisms of SC activity in the present study. Evidences for the secretion of neurotrophic factors by SC are inconsistent in the literature. However, in some publications, the presence of neurotrophic factors has been identified in peripheral nerve homogenates, there are reports in which the authors demonstrate the absence of these factors^52^,^53^,^54^,^55^,^56^. Based on our results, the assumption that the observed impact of grafted SC is due to neurotrophic factors remains questionable. Conversely, the lack of these factors did not limit the beneficial effect of the use of SC therapy for dying neurons, which suggests that the neurotrophic factors are not the only necessary stimulus for SC-dependent neuronal regeneration; it seems to be regulated in a more complex way^54^.

### Migration of intravitreally transplanted SC

Impaired migration of transplanted cells within the retinal structure is perceived as a basic problem of ocular cell therapies that results in reduced bioavailability of active agents secreted by the transplanted cells^13^,^14^,^57^,^58^. To improve the integration of transplanted cells *in vivo*, intravitreal injection of proteolytic enzymes, to digest retinal ILM, or suppression of glial cells (i.e., using intravitreal α-aminoadipic acid) was reported^14^. In both cases there was no increase in neuroprotective effect observed^14^. Cell migration in *ex vivo* conditions can be improved by mechanical breakdown of the ILM continuity through pipette tip trauma^13^,^59^. Our observations on SC migration and integration with the host retina are opposite to those presented by other authors. Based on *ex vivo* experiment with retinal explants, it might be expected that increasing of transplanted cells penetration into the host retina is not beneficial, because it disrupts the layered retinal structure, which may cause disintegration of the complex neural network. Additionally, it is important to note, the extra-retinal location of transplanted SC *in vivo* in our study was sufficient to ensure a satisfactory neuroprotective and pro-regenerative effect.

### Predominance of SC therapy

Different precursor cells indeed exhibited neuroprotective effects, but this was accompanied by many problems of a technical nature (e.g., the need for additional intraocular cell activation – erythropoietin in the case of Müller cell precursors and inflammatory stimulation in the case of oligodendrocytes precursors (*zymosan*), proteolysis of the retinal ILM, glial cell inhibition with glutamate analogues, immunosuppressive agents due to host versus graft immunoreactivity or uncontrolled proliferation and differentiation of transplanted cells) that make putative treatment difficult or even impossible to apply in clinical settings^2^,^11^,^12^,^14^. In our study, the RGC and optic nerve axon survival was similar to that observed in therapy with mesenchymal stem cells, and even better than that described for oligodendrocytes precursors and olfactory glial cells^10^,^12^,^25^. Therapy with predegenerated SC had an advantage over the cited types of progenitor cells because it did not require any additional treatments after transplantation.

### Different outcomes of SC transplantation in acute and chronic optic nerve damage

We observed differences in the neuroprotective activity of SC between the ONC and glaucoma model; in the second one (i.e., glaucoma), SC impact on RGC survival was more pronounced. We suspect that the explanation for this phenomenon might be associated with different follow-up times (i.e., 10 days vs 6 weeks). In the glaucoma model, longer incubation time in the vitreous potentially allowed transplanted SC to better adapt and was further beneficial to cell functions compared with the short duration in the ONC model. Another reason might be hidden in differing mechanisms of neuropathy (i.e., more acute in ONC) and localization of initial damage.

### SC transplantation prevents synaptic proteins impairment

Neuroplasticity in glaucoma has been described mostly with respect to brain structures; however, changes in retinal synaptic markers expression were reported with respect to ocular hypertension models^60^,^61^,^62^,^63^. Some authors reported that up to approximately the 14^th^ day of high-pressure exposure, synaptic proteins were overexpressed in retina, mostly within the IPL; then this expression dramatically decreased, which might be indirect evidence of the existence of natural pro-regenerative mechanisms that become gradually insufficient during the course of glaucoma^62^,^63^. In our study, a 6-week duration of ocular hypertension caused visible underexpression of synaptic markers within the retina in instances of untreated glaucoma and preservation of synaptic marker overexpression in SC-treated cases. This allowed us to conclude that SC therapy might prolong the functioning of natural defensive mechanisms that exist in retinal neurons and protect them against depression. Surprisingly, synaptic marker expression was also visible within the GCL and RNFL, which might suggests that SC therapy can enhance and extend neuronal web regulation, expressed in synaptic proteins synthesis and transport, however this direction would require further investigations^64^.

The novel therapy proposed in this study creates conditions to eliminate some of the identified barriers for ocular cells transplantation and allows us to observe direct neuroprotective and pro-regenerative effects in ongoing optic neuropathy without any additional modifications to transplanted cells after intravitreal injection. SC represent mature, differentiated cells, which is why almost no risk of uncontrolled proliferation exists. An additional advantage is that isolating and culturing them is a simple process^65^. Predegenerated Schwann cells might be isolated from each of the peripheral nerves, so they might be prepared and applied also as autotransplants; the best quality SC tend to originate from mixed (i.e., sensory-motor) nerves^53^,^54^. Based on the above description of predegenerated SC therapy, we demonstrated that it might be a promising, effective and easy to apply alternative cell therapy for glaucoma.

## Methods

### Animals

The study protocol was approved by the National Animal Experiment Board (Finland) and the Local Ethical Committee of the Medical University of Silesia (Poland). Experiments were conducted in accordance with the ARVO statement for the use of Animals in Ophthalmic and Vision Research. In all experiments, we used male Wistar rats (Experimental Medicine Center, Medical University of Silesia, Katowice, Poland and Laboratory Animal Center, University of Eastern Finland, Kuopio, Finland). For predegenerated Schwann cell culture, we included two 8-week-old rats, and for *ex vivo* experiments, we used four P2 rat pups and eight 4-week-old rats. For *in vivo* experiments we used fifty 40-week-old rats, 30 for the glaucoma model and 20 for the optic nerve crush model. Each *in vivo* group was divided into 2 equal subgroups, one to which we applied cell therapy and another to serve as a control group. Experimental *in vivo* procedures on eyes were performed unilaterally; the contralateral eye was kept intact as a healthy control. For animal anesthesia, we used an intraperitoneal injection of a mixture of ketamine (50 mg/kg; Ketalar, Pfizer Oy Animal Health, Finland; VetaKetam, VETAGRO, Poland) and medetomidine (0.4 mg/kg; Domitor, Orion Oy, Finland) or xylazine (5 mg/kg; Xylapan, Vetoquinol Biowet, Poland).

### Predegenerated SC culture

Animals for SC isolation were anesthetized; sciatic nerves were cut bilaterally at the level of the hip joint, and animals were maintained in standard conditions for 7 days with facilitated access to food and water. Thereafter distal stumps of transected sciatic nerves were collected. After collection of nerves, animals were sacrificed. The nerves were cultured on 12-well plates at 37 °C in humidified air with 5% CO_2_ in Endothelial Cell Growth Medium (EGM2MV BulleKit, Lonza, Basel, Switzerland) supplemented with 10 μg/ml insulin (Sigma, St. Louis, MO, USA), 12 ng/ml heregulin α (Sigma, St. Louis, MO, USA), 3 µM forskolin (Sigma, St. Louis, MO, USA) and 10 μg/ml pituitary bovine extract (Sigma, St. Louis, MO, USA) as described before^65^. The cells, after the third split, were double-stained with anti-S100 antibody (dilution 1:300) and anti-GFAP antibody (dilution 1:1000). The ratio of double immunostained cells was manually calculated from 5 visual fields under 40× magnification in relation to DAPI-positive nuclei. After characterization, SC were incubated with eGFP-coding lentiviral particles (LV-eGFP, CMV promoter, kindly donated by professor Seppo Ylä-Herttuala, Department of Biotechnology and Molecular Medicine, A.I. Virtanen Institute for Molecular Sciences, University of Eastern Finland, Kuopio). After the transduction procedure, cells were split, re-cultured twice and collected for intravitreal transplantation at a density of 1 × 10^6 ^cells in 5 μl of PBS and for *ex vivo* delivery at a density of 1 × 10^4 ^cells in 2 μl of PBS. For *in vivo* regeneration and neuroplasticity detection, non-transduced SC were used.

### SC proteomics by mass spectrometry

Medium samples and SC homogenate were processed according to Grønborg *et al*. and *Varjosalo et al*. with the authors’ modifications^66^,^67^. Briefly, samples were adjusted to a pH 8.0–9.0 with 1.0 M ammonium bicarbonate (Sigma, St. Louis, MO, USA). Tris (2-carboxyethyl) phosphine hydrochloride at a final concentration of 5 mM (Sigma, St. Louis, MO, USA) was used for the reduction of disulfide bonds at 37 °C for 30 minutes. Iodoacetamide 100 mM (Sigma, St. Louis, MO, USA) was used for blocking cysteine residues at RT in the dark for 30 minutes; thereafter, the samples were incubated with trypsin (Promega V5113, Madison,WI, USA) with an enzyme to substrate ratio of 1:20 (w/w) at 37 °C overnight. The trypsinized samples were purified with C18 silica microspin columns (The Nest Group, Southborough, MA, USA) and analyzed on a hybrid LTQ Orbitrap Elite Mass Spectrometer (Thermo Scientific, Rockford, IL, USA) coupled to an Easy nLCII (liquid chromatography) nanoflow system (Thermo Scientific, Rockford, IL, USA) via a nanoelectrospray ion source (Thermo Scientific, Rockford, IL, USA). The following solvents were used for liquid chromatography separation of the digested samples: solvent A (0.1% formic acid, 98% water, 2% acetonitrile), and solvent B (0.1% formic acid, 98% acetonitrile, 2% water). Peptides were eluted the analytical column with a gradient of 60 min of buffer B ranging from 5% to 35% at a constant flow rate of 300 nl/min followed by a 10 min gradient from 35% to 80% of solvent B.

As a positive control for the MS analysis, a solution of 1.0 and 0.1 ng/ml human brain derived neurotrophic factor (BDNF, PeproTech EC Ltd, London, UK) and 1.0 and 0.1 ng/ml rat ciliary neurotrophic factor (CNTF, PeproTech EC Ltd, London, UK) in Dulbecco’s Modified Eagle Medium: Nutrient Mixture F-12- (DMEM-F12, Sigma, St. Louis, MO, USA) was used.

### Proteomic data analysis

The RAW files were analyzed using Thermo Scientific™ Proteome Discoverer™ 1.4 software connected to the SEQUEST® search engine, constrained with a precursor mass tolerance of 10 ppm and fragment mass tolerance of 0.8 Da, respectively. Spectra were searched against the Protein Knowledgebase (UniProtKB reviewed completed *Rattus norvegicus* database) with the following parameters: enzyme specificity was set to Trypsin with up to 2 missed cleavages. Oxidation (+15.996 Da) of methionine was selected as a variable modification, carboxymethylated (+57.021 Da) cysteine was set as a fixed modification. Data were filtered with a peptide confidence set to <5% FDR. Proteins with at least one unique peptide were considered as identified. Automated annotation of identified proteins was completed with Gene Ontology (GO) classifications by Annotation®.

### Experimental glaucoma

After rats (n = 30) were anesthetized, ocular hypertension was induced as previously described, using “the bead model with initial high-pressure injury”, by intracameral injection of 5 μL of 6-μm-diameter beads (Polybead Microspheres; Polysciences, Inc., Warrington, PA, USA), 5 μL of 10-μm-diameter beads and 5 μL of viscoelastic solution (10 mg/mL sodium hyaluronate; Healon; Advanced Medical Optics Inc., Santa Ana, CA, USA) using a glass cannula with a 50-μm-diameter tip^68^. The right eye served as a healthy control. IOP measurements were obtained using a laboratory tonometer (TonoLab, Icare, Finland) three times during first week and then once a week. The rats were sacrificed 6 weeks after the beads were injected, except of 4 additional animals sacrificed 2 and 4 weeks after beads injections to evaluate transplanted SC localization. Retinas of 6 animals from each group were processed as whole mounted for stereology, other were used to prepare paraffin cross-sections.

### Optic nerve crush

Rats (n = 20) were anesthetized and optic nerve crush (ONC) was performed as described previously for mice with modifications according to the species^69^. The procedure was performed in a non-traumatic way, without cutting off the eyelid, which prevented eye surface scarification. The optic nerve was exposed via a conjunctival incision and crushed approximately 0.5 mm behind the globe for 10 seconds using a self-closing forceps to ensure reproducibility and constant force. The right eye served as a healthy control. The rats were euthanized 10 days after ONC. Retinas of 6 animals from each group were processed as whole mounted for stereology, other were used to prepare paraffin cross-sections.

### Intraocular delivery of Schwann cells

Cell allotransplantation was performed on the day after induction of ocular hypertension and the day after the ONC procedure. Injections were administered 1 mm posteriorly to the corneal limbus using a 5 μl Hamilton syringe combined with a 34 G needle. After injection, the needle was kept in the eye for approximately 2 minutes and area of the insertion wound was covered with antibiotic ointment (Oftan Chlora 10 mg/g, Santen Oy, Helsinki, Finland) to reduce the risk of infection and minimize the outflow of the cells.

### *Ex vivo* experiments

For the *ex vivo* study, we used 2 different models of retinal explant preparation and culture. To observe the direct impact of predegenerated SC on retinal neurite outgrowths, we prepared reversed retinal explants from newborn P2 rats (n = 4). To examine direct interactions between the GCL and SC, a co-culture of SC with retinal insert explants from 4 week-old rats (n = 8) was prepared.

### Insert retinal explants

Animals for insert explants preparation were sacrificed and their eyeballs were collected in an ice cold PBS solution containing 1% penicillin-streptomycin (Gibco, Carlsbad, CA, USA). Retinas were isolated from eyeballs, cut into 4 quadrants and placed on culture inserts (0.4 μm Millicell tissue culture insert, Millipore, Billerica, MA, USA) with GCL on the top, as previously described^13^. Inserts were placed in a 24-well plate containing culture medium consisting of Neurobasal A (Gibco, Carlsbad, CA, USA) supplemented with 5% fetal bovine serum (FBS), 2% B-27 supplement (Gibco, Carlsbad, CA, USA), 1% N2 supplement (Invitrogen, Carlsbad, CA, USA), 1% penicillin-streptomycin solution (Gibco, Carlsbad, CA, USA) and 0.4% GlutaMax (Gibco, Carlsbad, CA, USA). Thirty-two explants were cultured with 2 µl of SC/PBS suspension (approximately 104 cells per explant) placed on each explant surface. In 16 explants, we avoided contact between the pipette tip and the explant surface during placement of the cell suspension drop; another 16 explants received a SC suspension accompanied by slight contact of the pipette tip with the explant surface (to mechanically break the continuity of the retinal inner limiting membrane – ILM). As a control, we placed 2 μl of PBS on another 16 explant surfaces without and 16 with pipette tip contact. The medium was changed every second day. After 10 days of culture at 37 °C and 5% CO_2_, explants were fixed with 4% PFA overnight at +4 °C; 10 explants from each group were processed as whole mounted retinas for RGC counting using stereology software; cryosections of 6 explants from each group were processed for immunostainings.

### Reversed retinal explants

For reversed explants, rat pups were sacrificed; eyeballs were removed and stored in ice cold Neurobasal A medium (Gibco, Carlsbad, CA, USA) containing 1% penicillin-streptomycin solution (Gibco, Carlsbad, CA, USA). Retinas were isolated, cut in 4 round parts, and each part was placed on the bottom of a 24-well plate with down-orientated GCL, as described by Gaublomme *et al*.^70^. Before plating, wells were coated overnight at room temperature (RT) with 0.25 mg/ml poly-L-lysine (Sigma, St. Louis, MO, USA) in PBS followed by a 2 h-coating with 2 μg/ml laminin (Sigma, St. Louis, MO, USA) in PBS at 37 °C and 5% CO_2_. Explants were cultured in Neurobasal A medium (Gibco, Carlsbad, CA, USA) and supplemented as for insert explants; the medium was changed every day. Following this method, we prepared 30 explants: 10 of them were cultured in standard medium as a negative control; 10 explants were cultured as a positive control with 5 ng/ml of rat BDNF, a known inducer of neurite growth (Life Technologies, Carlsbad, CA, USA); and 10 were co-cultured with 10^3^ of predegenerated SC added into each well in parallel with explants plating. After 72 hours, explants were fixed with 4% PFA overnight at +4 °C and processed for immunostaining and neurites tracing.

### Optical Coherence Tomography (OCT) imaging

For OCT and fundus photographs, animals with transparent optic centers were selected. Scans were obtained from between 4–6 weeks of damage in glaucoma group and 3–10 days of damage in ONC group. Because satisfying optical center transparency was difficult to achieve after both applied procedures, there was a small number of animals in the subgroups (in the glaucoma group 2 animals with SC transplantation and 2 animals with PBS injection, in the ONC model 3 animals with SC transplantation and 3 animals with PBS injection). The procedure required general anesthesia and topical mydriasis with 0.5% tropicamide (Santen Oy, Helsinki, Finland). OCT scans and fundus photographs were obtained with HRT Spectralis OCT (Heidelberg Engineering, Germany). Photographs of the eye fundus as well as retinal scans in the parapapillary area were taken with a focus =32 dptr. We measured the following parameters: optic nerve disc, cup, and neuroretinal ring size, cup/disc ratio, retina and retinal nerve fiber layer (RNFL) thickness in the central and peripheral retina.

### Animal sacrifice

Rats were transcardially perfused under deep anesthesia with 0.9% NaCl for 2 min, followed by perfusion with 4% paraformaldehyde/0.1 M PBS, pH 7.4, for 10 min. Eyes were enucleated while preserving retrobulbar optic nerve stumps and post-fixed in 4% paraformaldehyde overnight at +4 °C; they were then washed in PBS overnight and processed for immunohistochemistry.

### Immunohistochemistry

Optic nerves were cut off behind the eyeball and divided into 2 stumps. Proximal stumps with unmyelinated nerve fibers were processed for immunostainings; distal stumps containing myelinated nerve fibers were used for axon counting. The total number of cells in the GCL was estimated in whole mounted retinas; paraffin cross-sections of eyeballs were used for immunostainings. For visualization, we used a fluorescent microscope, Zeiss Axio Scope. A1 (Zeiss, Oberkochen, Germany).

### Paraffin-embedded retinal sections

After fixation, eyeballs were embedded in paraffin according to a standard protocol, sectioned at 5 μm, and prepared for immunostaining by deparaffinization, rehydratation and blocking in 10% NGS 0.1% Triton X-100 in 0.05 M TBS for 30 min. Primary antibodies incubation was performed overnight at +4 °C (Supplementary Table 2). After each primary antibody samples were washed in 0.05 M TBS, pH 7.4 and incubated for 3 h at room temperature (RT) with an appropriate secondary antibody and washed again. To counterstain nuclei, samples were incubated with 1:10 000 dilution of 4′,6-diamidino-2-phenylindole (DAPI, Sigma-Aldrich, St. Louis, MO, USA) for 10 min at RT and mounted with Mowiol (Calbiochem, CA, USA).

### Optic nerves and *ex vivo* explant cryosections

After overnight fixation in 4% PFA at +4 °C, optic nerves and retinal explants were embedded in an optimal cutting temperature compound (Sakura Finetek Europe BV, Netherlands), sectioned at 10 μm, and processed for immunostaining as described earlier.

### Whole mounted retinas and whole mounted *ex vivo* explants

After fixation, floating samples were washed in PBS overnight in +4 °C and incubated in 20% NGS 0.1% Triton X-100 in 0.05 TBS for 45 min, and stained as described earlier.

### Retinal reversed *ex vivo* explants

After fixation, explants were incubated in 20% NGS 0.1% Triton X-100 in 0.05 TBS for 45 min. Primary antibodies were applied for 3 h at RT followed by a 3 h incubation at RT with a secondary antibody. Nuclei were counterstained with 4′,6-diamidino-2-phenylindole (DAPI, Sigma-Aldrich, St. Louis, MO, USA) for 10 min.

### TUNEL assay kit

To visualize apoptotic cells, we performed TUNEL staining on cryosections using a 30 minute-incubation with 0.1 M Tris-HCl, pH = 7.5/3% BSA/20% NGS followed by a 1 h-incubation with an *in situ* cell death detection kit at 37 °C (Roche, Mannheim, Germany). Nuclei were counterstained with 4′,6-diamidino-2-phenylindole (DAPI, Sigma-Aldrich, St. Louis, MO, USA) fluorescent stain (1:10 000 in 0.05 M TBS) for 10 minutes and mounted with Mowiol (Calbiochem, CA, USA).

### Optic nerve processing for axonal counts

To evaluate optic nerve axon damage, optic nerve cross-sections (n = 5 per group) were analyzed using stereological methods. After fixation in 4% paraformaldehyde, optic nerves were post-fixed in 1% osmium tetroxide for 30 minutes, dehydrated in a graded ethanol series and stained with 1% uranyl acetate in absolute ethanol for 1 hour. After staining, optic nerve stumps were infiltrated with epoxy resin at 60 °C for 48 hours, and then, 1 μm thick cross-sections of the optic nerve were cut and stained using toluidine blue.

### Stereology

The stereological software Stereo Investigator (MicroBrightField Inc, VT, USA) was used to count the total number of GCL cells in whole mounted retinas from *in vivo* experiments, estimate the RGC density in whole mounted retinal *ex vivo* insert explants, and count the total number of axons in optic nerve sections. To evaluate neurite outgrowth parameters in reversed P2 retinal explants, we performed neurite tracing using the stereology software Neurolucida (MicroBrightField Inc, VT, USA). Measurements included the number of neurites, their total and mean length [μm], their surface area [μm^2^], and their arboration rate [%].

### Statistics

Statistical analysis was performed with the IBM statistical software SPSS v. 20. Descriptive statistical results were reported as the mean ± standard deviation (SD). A Kolmogorov–Smirnov test was used to check whether the data were normally distributed. Comparisons between groups were performed using a Mann-Whitney U-test, paired samples Wilcoxon test or paired samples t-student test. *P* values < 0.05 were considered statistically significant.

## Additional Information

**How to cite this article**: Smedowski, A. et al. Predegenerated Schwann cells – a novel prospect for cell therapy for glaucoma: neuroprotection, neuroregeneration and neuroplasticity. *Sci. Rep.* **6**, 23187; doi: 10.1038/srep23187 (2016).

## Supplementary Material

Supplementary Information

Supplementary Table 1
