# Supplementary material for: Predegenerated Schwann cells–a novel prospect for cell therapy for glaucoma: neuroprotection, neuroregeneration and neuroplasticity
Source: Sci Rep. 2016 Apr 1;6:23187. doi: 10.1038/srep23187 (PMC4817039; doi:10.1038/srep23187)
Supplement: Supplementary Information [file srep23187-s1.pdf]

Supplementary Informations

Predegenerated Schwann cells – a novel prospect for cell therapy for glaucoma: neuroprotection, neuroregeneration and neuroplasticity

Smedowski Adrian[1,2,3], Liu Xiaonan[4], Pietrucha-Dutczak Marita[1], Matuszek Iwona[1], Varjosalo Markku[4], Lewin-Kowalik Joanna[1,*]

[1]Chair and Department of Physiology, School of Medicine in Katowice, Medical University of Silesia, Medykow 18, 40-752 Katowice, Poland

[2]Department of Ophthalmology, University of Eastern Finland, P.O. Box 1627, 70211 Kuopio, Finland

[3]Department of Ophthalmology, School of Medicine with the Division of Dentistry in Zabrze, Medical University of Silesia, Panewnicka 65, 40-760 Katowice, Poland

[4]Institute of Biotechnology, P.O. Box 65, University of Helsinki, 00014 Helsinki, Finland

*Professor Joanna Lewin-Kowalik MD, PhD; Department of Physiology, Medykow 18, 40-752 Katowice, Poland; Phone: +48322525087, Fax: +48322088300, email: jlk4@wp.pl

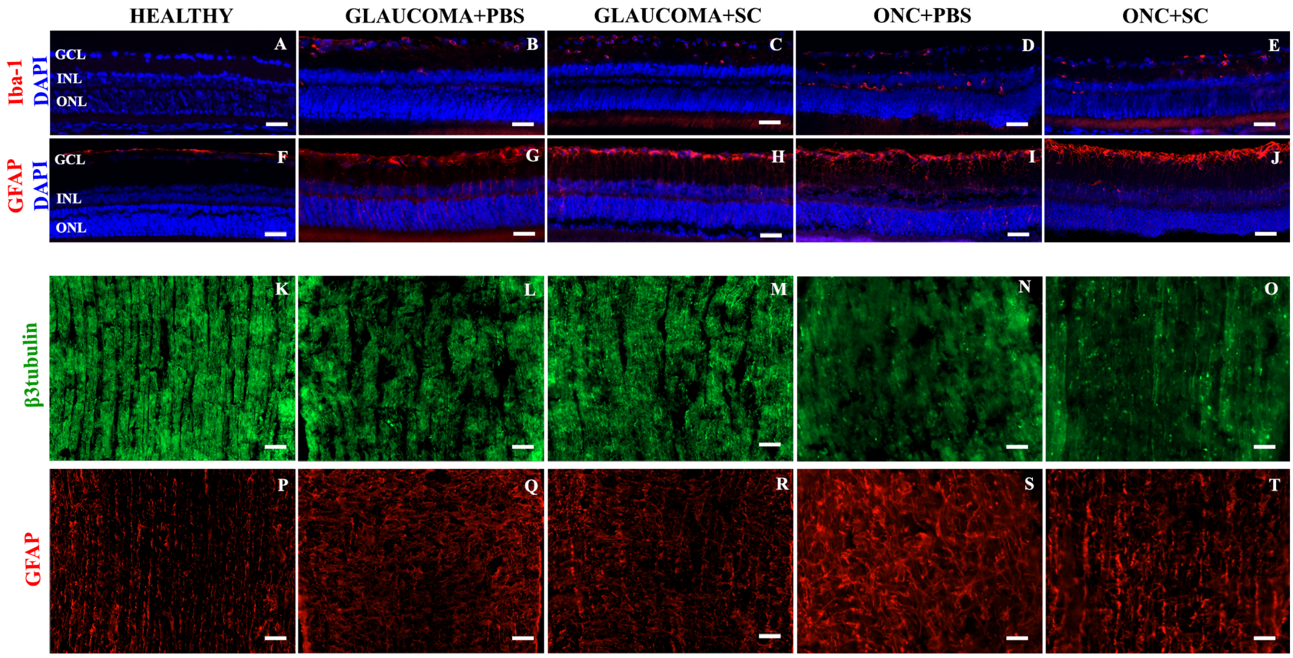

Supplementary Figure 1. Retinal cross-sections (A-J) and optic nerve longitudinal sections (K-T) with immunofluorescent staining. Within retina, applied cell therapy does not exacerbate microglial cells/macrophages infiltration (A-E) and retinal glia proliferation (F-J). In optic nerve longitudinal sections, SC therapy preserves lineal architecture and improved survival of RGC axons (K-O), as well as reduces reactive gliosis (P-T). Scale bar=50 μm. SC - Schwann cells; GCL - ganglion cell layer; INL - inner nuclear layer; ONL - outer nuclear layer; ONC – optic nerve crush.

Supplementary Table 1. Protein list identified in MS analysis of SC culture medium and SC homogenate. SC secretome and SC homogenate do not contain neurotrophic factors. Protein list includes medium analysis, SC homogenate analysis and positive controls samples (i.e., 1.0 ng/ml and 0.1 ng/ml of BDNF and CNTF, respectively). Attached as separated excel file.

Supplementary Table 2. Primary antibodies applied for immunostainings.

**Antibody**	**Manufacter**	**Dilution**	**Target**
mouse anti-NeuN (clone A60) MA B377	Millipore	1:500	neuronal nuclei
rabbit anti-β3-tubulin XP™ D7169	Cell Signaling Technologies	1:300	retinal ganglion cells
rabbit anti-GFAP Z0334	DAKO	1:1000	glial cells, astrocytes, Schwann cells
rabbit anti-GAP43 AB5220	Millipore	1:300	differentiating and regenerating neurons during axonal growth
rabbit anti-S-100 Z0311	Cell Signaling Technologies	1:300	Schwann cells, Müller cells
mouse anti-synaptophysin MAB329	Millipore	1:300	small presynaptic vesicles
rabbit anti-Iba-1 #019-19741	Wako	1:300	microglial cells, macrophages
